# Clinical Predictors of Progression From Metabolic Dysfunction-Associated Steatotic Liver Disease to Cirrhosis: A Narrative Review

**DOI:** 10.7759/cureus.91628

**Published:** 2025-09-04

**Authors:** Jane M Abucha, Mekdes M Wollel, Nethra Somannagari, Alousious Kasagga, Aayam Sapkota, Gichin Changaramkumarath, Lubna Mohammed

**Affiliations:** 1 Medicine, American University of Integrative Sciences, Bridgetown, BRB; 2 Pediatrics and Child Health, University of Gondar, Gondar, ETH; 3 Neurology, Gandhi Medical College, Hyderabad, IND; 4 Pathology, Peking University, Beijing, CHN; 5 Pathology, California School of Podiatric Medicine, Oakland, USA; 6 Internal Medicine, Chongqing Medical University, Chongqing, CHN; 7 Principles and Practice of Clinical Research, Harvard School of Public Health, Boston, USA

**Keywords:** cirrhosis of the liver, fibrosis, genetic predictors of liver disease, liver cirrhosis, metabolic dysfunction-associated steatohepatitis (mash), non-alcoholic fatty liver disease (nafld), non-alcoholic steatohepatitis (nash), risk factors for non-alcoholic fatty liver disease

## Abstract

Metabolic dysfunction-associated steatotic liver disease (MASLD) continues to affect many people around the globe and may increase the likelihood of liver-related morbidity and mortality. Early identification of individuals at risk for progression to liver cirrhosis is crucial to the mitigation of the disease effects. This review aims to summarize the current evidence on clinical factors predicting MASLD progression to cirrhosis.

A comprehensive literature search was conducted in PubMed, Cochrane, Medscape, JAMA, and Scopus covering studies from May 30, 2010, to May 30, 2025. Keywords included NAFLD, NASH, MASH, cirrhosis, risk factors, fibrosis, and progression.

Key predictors identified include metabolic syndrome components (obesity, diabetes, and dyslipidemia), genetic variants such as Patatin-like phospholipase domain-containing protein 3 (PNPLA3), transmembrane 6-superfamily member 2 (TM6SF2), Ras-related protein 14 family (RAB14), lifestyle factors (sedentary behavior, high-fructose diet), demographic traits (age, sex, and ethnicity), and histological markers (fibrosis stage, non-alcoholic steatohepatitis (NASH) features). Non-invasive detection tools such as the Fibrosis-4 (FIB-4) index, non-alcoholic fatty liver disease (NAFLD) fibrosis score (NFS), enhanced liver fibrosis (ELF), and perilipin-2 (PLIN2) and elastography are valuable tests for early identification.

A combination of metabolic, genetic, lifestyle, and histological factors predicts MASLD progression. Early detection and intervention are critical to prevent cirrhosis. There is a need for more standardized non-invasive tests and their availability.

## Introduction and background

Introduction

Metabolic dysfunction-associated steatotic liver disease (MASLD) continues to be the leading cause of chronic liver disease globally, with an estimated prevalence of approximately 25% of the world’s population [1]. MASLD was formally known as non-alcoholic fatty liver disease (NAFLD). Most recent data point to an increase in this number to approximately 32% due to the increase in obesity [2]. MASLD is a broad category of liver diseases, from mild to severe forms of MASLD, and has become a severe condition called non-alcoholic steatohepatitis (NASH). NASH is characterized by hepatocellular injury and inflammation that may result in progressive fibrosis, cirrhosis, and hepatocellular carcinoma over time [3]. The causes of NASH that stem from metabolic syndromes are now referred to as metabolic dysfunction-associated steatohepatitis (MASH) [4]. As stated by reference [3], MASH is a progressive liver disease linked to complications related to the liver and its mortality. According to reference [5], MASH is defined as a chronic, progressive liver disease marked by fat accumulation, inflammation, and the potential for fibrosis. This heightens the risk of cirrhosis, liver failure, and liver cancer [5].

Researchers in reference [6] defined MASH as a long-term liver condition that ranged from uncomplicated steatosis to steatohepatitis and fibrosis, which may progress to cirrhosis. One of the emerging predictors of NAFLD is the increase in obesity and metabolic syndrome that comes with it as the world population lives longer. As obesity, type 2 diabetes mellitus (T2DM), and metabolic syndrome continue to rise worldwide, the burden of MASLD is expected to increase, posing significant public health challenges [7]. Despite its growing prevalence, the natural history of MASLD remains incompletely understood, particularly regarding the rates and determinants of progression to cirrhosis.

One major cause of liver cirrhosis is alcoholic liver disease (ALD), and it is often complicated by portal hypertension and liver failure [8]. The progression pathway of MASLD is influenced by metabolic factors over time [4,9]. MASLD progression rates have been steadily increasing due to diabetes and age and posing challenges to healthcare providers.

The risk factors that increase the progression rates of MASLD are of paramount importance. A clear guideline informs clinical decision-making, screening, resource allocation, and public health strategies. Identifying predictors of liver disease progression, including clinical features and genetic predispositions such as Patatin-like phospholipase domain-containing protein 3 (PNPLA3) polymorphisms [10,11], is overdue. This includes lifestyle factors that are critical for preventing MASLD liver-related complications and mortality. Current available studies on NAFLD progression have reported fibrosis progression in simple steatosis and NASH to be between four and seven years [12]. Causes from metabolic and non-alcoholic-related risk factors were found to be a burden through the estimation of prevalence [13]. In this review, we focus on NAFLD progression and non-invasive assessment tools (NITs) [10].

This narrative review aims to synthesize current evidence on the rates of progression to cirrhosis in MASLD, providing a comprehensive assessment of key determinants and highlighting gaps in knowledge that warrant further investigation. The review also explores the key clinical, genetic, and lifestyle factors contributing to the transition from MASLD to cirrhosis. In addition to analyzing these risks, our findings aim to inform healthcare practitioners such as hepatologists, primary care providers, and policymakers in designing effective strategies for MASLD-related liver disease prevention and management.

A short version of the current article has been published by the Arizona State Board of Nursing under Nursing Network in July 2025 by Jane Abucha [14].

Methods

We conducted a narrative review using PubMed, JAMA, Cochrane database, Medscape, and Scopus sources. Articles published between May 30, 2010, and May 30, 2025, were included. Search terms were "NAFLD," “NASH, MASH,” "liver cirrhosis," "risk factors," "fibrosis," and "progression." Both observational studies and meta-analyses were included. The exclusion criteria were for studies focusing exclusively on ALD or pediatric populations. Other studies excluded were studies that the researchers were not able to use because they did not have open access.

Epidemiology of NAFLD and cirrhosis

MASLD affects about 25% of the global population, with the highest prevalence in the Middle East and South America [15] and up to 32% according to researchers [2] in recent studies. Cirrhosis represents the end stage of progressive fibrosis and is associated with significant morbidity and mortality. MASH, which is a subtype of MASLD, remains progressive and is often linked to PNPLA3, specifically gene variant rs738409 encoding for an isoleucine to methionine substitution at position I148M (Table 1) [16].

**Table 1:** Key clinical predictors of metabolic dysfunction-associated steatotic liver disease (MASLD) progression to cirrhosis

Category	Key predictors	Sources
Metabolic	Obesity, type 2 diabetes, dyslipidemia, insulin resistance	References [3,17]
Genetic	Patatin-like phospholipase domain-containing protein 3 (PNPLA3) I148M variant, transmembrane 6-superfamily member 2 (TM6SF2) variant	References [18,19]
Lifestyle	Sedentary behavior, high-fructose diet, low physical activity	References [15,20]
Demographic	Older age, male sex, Hispanic ethnicity	Reference [20]
Histological	Fibrosis stage, presence of non-alcoholic steatohepatitis (NASH) (ballooning, inflammation)	Reference [17]
Detection tools	Fibrosis-4 (FIB-4) index, non-alcoholic fatty liver disease (NAFLD) fibrosis score (NFS), transient elastography	Reference [3]

## Review

Literature review and risk factors for progression

MASLD has emerged as a major public health concern, affecting approximately 25% of the global population [15]. The disease spectrum ranges from simple steatosis to NASH, which can progress to fibrosis and ultimately cirrhosis. MASH, a subtype of MASLD, is progressive, with potential for fibrosis and liver cirrhosis [21]. Multiple studies have investigated the clinical factors that predict the progression of MASLD to cirrhosis, as detailed in the subsequent paragraphs [22].

Metabolic syndrome components are consistently identified as the primary drivers of MASLD advancement. Reference [3] documented and emphasized the roles of the major risk factors, which are obesity, type 2 diabetes, and dyslipidemia, in accelerating liver fibrosis. Their guidance highlights that patients with diabetes are at particularly high risk, with nearly double the progression rates compared to non-diabetics. Moreover, insulin resistance has been shown to directly contribute to hepatic steatosis and inflammation, further promoting fibrosis [17].

Genetic predispositions also play a crucial role and are linked to increased liver fat and fibrosis onset. As stated in reference [16], the variants of the PNPLA3 gene, specifically the rs738409 variant that results in an isoleucine to methionine change at position I148M, are significantly linked to fibrosis. The PNPLA3-I148M variant is a key driver of hepatic steatosis, inflammation, and fibrosis [16]. This genetic predisposition leads to increased fat content and fibrosis progression, independent of metabolic risk factors [19]. In a similar vein, variants of the transmembrane 6-superfamily member 2 (TM6SF2) have been associated with increased stages of fibrosis and the severity of NASH [18]. AZD2693 genetic marker is a liver-targeted antisense oligonucleotide (ASO) designed to reduce the expression of the PNPLA3 variant [16]. These genetic markers are becoming valuable tools in risk stratification models.

Lifestyle and demographic factors further modulate the disease risk. Sedentary behavior and many other high-fructose diets, as well as low physical activity levels, are associated with worse liver outcomes, as researchers found [15]. Furthermore, advanced age, male gender, and Hispanic ethnicity have been recognized as demographic factors associated with increased rates of progression [1,20].

Histological studies remain the gold standard for assessing progression risk as well as determining its effects and time in the body. As stated in reference [17], the stage of cirrhosis serves as the most significant predictor of liver-related morbidity and mortality among patients with progressive NASH. Notably, the presence of ballooning degeneration and lobular inflammation is noted as a hallmark of NASH and significantly accelerates fibrosis progression [22].

Non-invasive detection strategies are gaining more prominence in clinical practice today than before, as the number of new NASH/MASH cases continues to rise globally. Tools such as the perilipin-2 (PLIN2) and Ras-related protein 14 family (RAB14) are monocyte-based non-invasive markers that have shown promise in predicting MASH [10]. PLIN2 is a lipid droplet-associated protein, and its primary function is to regulate lipid storage and metabolism. It is expressed in metabolically active tissues such as the liver and adipose tissues. In the hepatocytes, high PLIN2 is associated with steatohepatitis.

RAB14 regulates transport between early endosomes and the trans-Golgi network and helps control the intercellular trafficking of glucose transport 4 (GLUT4) in adipocytes and muscles. RAB14 is linked to cancer progression through the altered trafficking of growth receptors. A decrease in PLIN2 is observed when used for monitoring resolutions, but an increase in RAB14 shows improvement [10].

The Fibrosis-4 (FIB-4) index and NAFLD fibrosis score (NFS), serum fibrosis markers, and transient elastography have demonstrated utility in identifying patients with advanced fibrosis [3]. The FIB-4 index is a non-invasive, cost-effective tool that has been in use to assess the likelihood of fibrosis. This simple test is calculated from a blood test performed in a comprehensive metabolic panel or liver panel. The FIB-4 index is useful as a first-line screening tool by primary care providers with potential for referral to specialists for further evaluation. According to reference [6], the FIB-4 index has utility in early screening, but it is neither sensitive enough nor accurate enough to detect early fibrosis. The FIB-4 index also has false positives in certain conditions, such as in those who use alcohol and persons with thrombocytopenia. Therefore, the FIB-4 index should be used in conjunction with other non-invasive tools to generate a comprehensive analysis of a person’s condition to allow clear clinical practice decision-making [6].

The enhanced liver fibrosis (ELF) test is another non-invasive blood test used to assess the severity of liver fibrosis in patients with MASLD and other chronic liver diseases [21]. The ELF test has demonstrated the ability to predict liver cirrhosis, liver failure, and liver cancer accurately among persons with MASLD [21]. Sanyal et al. further concluded that the test can identify those at high risk of progression to cirrhosis and is comparable to the gold standard biopsy in some research. These non-invasive methods offer a safer and more accessible alternative to liver biopsy. They support early intervention and management, preventing major, cumbersome outcomes and their complications in life [23]. Although non-invasive tests hold promise for monitoring MASLD and liver fibrosis, they have some limitations in the early stages of the disease [10]. The same limitations apply to monitoring resolutions (Table 2).

**Table 2:** Summary of the non-invasive tests used both in practice and in research, the methods used, and the invasiveness and accuracy of the test AST: aspartate transaminase; ALT: alanine transaminase; ELF: enhanced liver fibrosis

Test & citation	Usage	Method	Invasiveness	Accuracy
PLIN2 [10]	Biomarker for hepatic steatosis & lipid accumulation in metabolic dysfunction-associated steatotic liver disease (MASLD)	Measured in blood or tissue by biopsy. Only in research	Mild or non-invasive if by blood, highly invasive if by biopsy	Not yet validated as a stand-alone test. Mostly used in research
Ras-related protein 14 family (RAB14) [10]	Investigational biomarker in lipid metabolism and vesicle trafficking in MASLD	Measured in tissue cells	Highly invasive if assessed by biopsy	Currently used in the research stage, not a standard diagnostic tool yet
Fibrosis-4 (FIB-4) index [6]	Clinical scoring tool to estimate liver fibrosis in patients with MASLD and some viral hepatitis	Blood tests from AST, ALT, and platelets	Mild or non-invasive	Sensitivity ~70%-80% for advanced fibrosis. Specificity > 85% when using high cut-offs
PNPLA3 [7]	Genetic test for predicting MASLD and non-alcoholic steatohepatitis (NASH) to fibrosis and cirrhosis	Saliva or blood test	Non-invasive	High predictive value in genetic models. Not used as a stand-alone test
AZD2693 [7]	Investigational drug targeting the PNPLA3 pathway to treat NASH in patients with Patatin-like phospholipase domain-containing protein 3 (PNPLA3) variant I148M	Injections	Minimally invasive by injections	It is not a diagnostic test
ELF test [21]	To assess the severity of fibrosis in metabolic-associated steatohepatitis (MASH)	Blood	Mild	~0.80%-0.90% for advanced fibrosis and has outperformed FIB-4 index in some cohort studies

Discussion

Current evidence highlights the multifactorial nature of MASLD progression to NASH and MASH, then to liver cirrhosis. Metabolic dysfunction, genetic susceptibility, lifestyle choices, and histological severity all interplay in disease advancement [3]. The genetic drivers are the most important parts of the puzzle in progression. The PNPLA3 I148M variant is a key driver of hepatic steatosis, inflammation, and fibrosis [11]. PLIN2 and RAB14 are monocyte-based non-invasive markers that have shown promise in predicting MASH and fibrosis severity and resolution after bariatric surgery. The FIB-4 index and NFS are also essential and have been in use; however, they have limited ability to identify MASLD in patients with advanced fibrosis and may give false-positive results in older people. While biopsy remains the gold standard, non-invasive tools are increasingly reliable and important for follow-up [4].

Metabolic syndrome related to MASH

Metabolic syndrome plays a pivotal role in the pathogenesis and progression of MASH, formerly known as NASH, to advanced liver fibrosis and cirrhosis [3]. This is a multifactorial syndrome and comes with multiple constellations and risks [24]. These include central obesity, dyslipidemia, and insulin resistance that eventually becomes chronic inflammation and fibrogenic state, and further accelerates to cirrhosis [3]. Insulin resistance is an important player in promoting hepatic steatosis by increasing free fatty acid influx to the liver for lipogenesis while impairing lipid export [25]. Lipid imbalance is a key promoter of oxidative stress and mitochondrial dysfunction and activates stellate cells to fibrosis.

Type 2 diabetes has been shown in multiple cohort studies to be an independent predictor of advanced fibrosis and cirrhosis. With the increase in obesity, systemic inflammation from metabolic syndrome also contributes to cardiovascular morbidity, which remains the leading cause of death in the United States [13]. This is the major reason that individuals with these conditions may have a high risk of developing risk factors for NASH/MASH.

Genetic factors that are key drivers of NAFLD progression to cirrhosis

Genetic predisposition is definitely a risk factor that plays another central role in determining the progression of NAFLD to MASH and eventually cirrhosis. PNPLA3 is the most extensively studied gene, particularly the rs738409 C & D variants, which encode for I148M substitution [19]. The polymorphism of this gene is consistently associated with increased hepatic fat accumulation, inflammation, and fibrosis. The variant with the G allele appears to impair triglyceride hydrolysis in hepatocytes, promoting the retention of lipid droplets and lipotoxicity. This is the reason that the G allele has a higher risk of developing advanced fibrosis and cirrhosis [19].

Other genetic drivers include TM6SF2 and membrane-bound O-acyltransferase domain-containing 7 (MBOAT7) [11]. TM6SF2 variant E167K reduces hepatic lipid export and leads to the exacerbation of steatosis and fibrosis. The variants MBOAT7 are linked to altered phospholipid remodeling and increased inflammation, promoting liver injury. New data show that the role of hydroxysteroid 17-beta dehydrogenase 13 (HSD17B13) variant rs72613567 in hepatic lipid droplet protein is protective [11]. The loss-of-function mutation of this variant is protective from fibrosis.

Genetic screening for the HSD17B13 variants may help identify those persons at risk or with a more favorable prognosis and target them with future therapies [16]. Clinical implications could be a way to find drugs that inactivate this variant to protect those at risk from fibrosis.

Lifestyle factors predisposing to MASLD

Lifestyle factors play a role in the onset and progression of MASLD and may intensify the advanced stages of MASH and cirrhosis at the end [1]. Sedentary behavior and poor diets with high saturated fats and sugar are associated with metabolic syndrome as well as obesity [26]. They are exacerbated by physical inactivity and impaired lipid metabolism. Smoking excessively and alcohol consumption, poor sleep, and psychosocial stress can synergistically worsen liver injury in those already at risk [3]. These factors create a state of chronic inflammation, which can potentially become a factor for fibrosis [5].

Histological factors

The key factors in histology are the degree of hepatocellular injury. These include the degree of hepatocellular ballooning, lobular inflammation, and stages of fibrosis (1-4) [15]. NASH is characterized by inflammation and ballooning and is predictive of fibrosis advancement [21]. Bridging fibrosis is strongly correlated with the risk of cirrhosis and poor outcomes [27]. Research also shows that portal inflammation and peri-sinusoidal fibrosis in zone 3 of the hepatic acinus are associated with a worse prognosis [14]. The gold standard for determining NASH and the course of liver disease progression is liver biopsy, which remains necessary. Histological features are associated with mortality in MASLD patients, and an accurate evaluation of the disease remains critical for staging, guiding treatment, and implementing long-term population risk prevention strategies [28].

Non-invasive tests

Non-invasive tests such as the NFS, ELF test, FIB-4 index, transient elastography (FibroScan), shear-wave ultrasound, and magnetic resonance elastography (MRE) are essential in the early detection of MASLD and its progression [3]. Liver biopsy is invasive and not suitable for routine monitoring, which necessitates the need for accurate non-invasive tests to identify and assess MASH severity and guide treatment response.

The NFS is used to estimate the risk of advanced liver fibrosis in patients with MASLD [26]. It is scored using a formula with a score < -1.455 to be low risk, -4.55-0.676 to be moderate risk, and >0.676 to be high risk of advanced fibrosis [26]. It is simple and inexpensive and uses regular blood samples, age, and body mass index (BMI). It also has a good negative predictive value (NPV) for advanced fibrosis and can help reduce the need for biopsy once it is negative [26]. It is often used with other tests to increase accuracy, and it is also less accurate in young people.

The FIB-4 index is cost-effective and widely available to be used in clinical practice from blood samples. Scores below 1.3 indicate low risk, 1.3-2.67 is moderate risk, and scores greater than 2.67 are at high risk for advanced fibrosis [6]. The FIB-4 index is useful for ruling out, but its limitation is also due to accuracy reasons. This added potential of non-invasive tests improves risk stratification and prognosis in patients with NASH/MASH and advanced fibrosis, resulting in improvement of clinical decision-making and reliance on such tools [6].

The ELF test has demonstrated the ability to predict liver-related clinical events [21]. These include, but are not limited to, cirrhosis, liver failure, and liver cancer among patients with MASH and advanced fibrosis. This means that the ELF test can identify those at high risk of progression to cirrhosis and is comparable to the gold standard biopsy in some research [21]. The ELF test is very promising and has shown the ability to detect fibrosis and offers a valuable non-invasive alternative to biopsy, reducing the need for liver biopsy.

All these tools are useful in monitoring and guiding referrals to specialists; however, factors like inflammation, congestion, and operator variability can affect accuracy [4]. Gaps remain in understanding the combined predictive power of these non-invasive tools, reliability, and factors associated with accuracy. Current and ongoing research is geared toward refining the broader use of non-invasive tests, especially in primary care and rural settings where access to liver biopsy or advanced imaging is limited (Table 3).

**Table 3:** Histological and non-invasive scoring tools

Tool	Score for advanced fibrosis	Risk or grade
Fibrosis-4 (FIB-4): <1.3 [3,6,17]	Low risk	Low risk
FIB-4: 1.3-2.67	Intermediate	Moderate risk, consider further evaluation for diagnosis
FIB-4: >2.67	High risk for advanced fibrosis	Refer to hepatology for liver biopsy
NAFLD fibrosis score (NFS):	Low risk	Advanced fibrosis is unlikely
NFS: -1.455 to 0.676	Intermediate risk	Further testing required
NFS: >0.676	High risk for advanced fibrosis	Refer to hepatology, likely advanced fibrosis
Enhanced liver fibrosis: <7.7 [17,21]	Low risk	Advanced fibrosis is unlikely
Enhanced liver fibrosis: 7.7-9.8	Intermediate risk	Consider further testing
Enhanced liver fibrosis: >9.8	High risk for advanced fibrosis	Refer to hepatology, likely advanced fibrosis
Transient elastography (FibroScan): <7.0 [17,21]	Low risk (F0-F1)	Low risk and fibrosis is unlikely
Transient elastography (FibroScan): 7.0-8.1	Intermediate to significant risk (F2)	Consider clinical correlation and further tests
Transient elastography (FibroScan): 8.2-9.6	Advanced or increased risk (F3)	Increased risk, refer to hepatology
Transient elastography (FibroScan): >9.6	Cirrhosis (F4)	High risk for cirrhosis, refer to hepatology
Magnetic resonance elastography (MRE): <2.5 [17,21]	Low risk (F0-F1)	Fibrosis is unlikely
MRE: 2.5-3.6	Intermediate risk (F2)	Intermediate risk, correlate clinically [17]
MRE: 3.6-4.0	Advanced fibrosis (F3)	Increased risk for advanced fibrosis, refer to hepatology [17]
MRE: >4.0	Cirrhosis (F4)	High risk for advanced cirrhosis, refer to hepatology [17]

## Conclusions

This review synthesized current literature and studies examining the progression of NAFLD to liver cirrhosis, with particular focus on the rates of progression and contributing factors. Eligible studies were selected based on clearly defined inclusion criteria. Data extraction was performed by one researcher and verified independently by a volunteer to ensure accuracy. This review highlights the current known theories of the progression of NAFLD to cirrhosis. The review found that the progression is multifactorial and continues to be driven by multiple factors, such as metabolic dysfunction comorbidities, genetics, lifestyles, and the histopathological features seen.

Early fibrosis detection remains essential with liver biopsy as the gold standard; however, because it is invasive, it may not be possible for repeat biopsies. Non-invasive tools such as elastography and serum markers have shown some promise and should be utilized. There is limited access to diagnostics in some areas, especially underserved areas, which continues to be a barrier. Further research should evaluate non-invasive tools across diverse populations, clarify monitoring strategies, and define the role of advanced practice providers in rural areas. The need to expand scalable non-invasive diagnostics and preventive strategies is crucial to reducing NAFLD-related cirrhosis globally.
